# Remote sensing and human health: new sensors and new opportunities.

**DOI:** 10.3201/eid0603.000301

**Published:** 2000

**Authors:** L R Beck, B M Lobitz, B L Wood

**Affiliations:** California State University, Monterey Bay, California 94035-2424, USA. lrbeck@gaia.arc.nasa.gov

## Abstract

Since the launch of Landsat-1 28 years ago, remotely sensed data have been used to map features on the earth's surface. An increasing number of health studies have used remotely sensed data for monitoring, surveillance, or risk mapping, particularly of vector-borne diseases. Nearly all studies used data from Landsat, the French Système Pour l'Observation de la Terre, and the National Oceanic and Atmospheric Administration's Advanced Very High Resolution Radiometer. New sensor systems are in orbit, or soon to be launched, whose data may prove useful for characterizing and monitoring the spatial and temporal patterns of infectious diseases. Increased computing power and spatial modeling capabilities of geographic information systems could extend the use of remote sensing beyond the research community into operational disease surveillance and control. This article illustrates how remotely sensed data have been used in health applications and assesses earth-observing satellites that could detect and map environmental variables related to the distribution of vector-borne and other diseases.


### Remote Sensing and Human Health: New Sensors and New Opportunities

**Louisa R. Beck,*† Bradley M. Lobitz,† and Byron L. Wood†**
California State University, Monterey Bay, California, USA;
NASA Ames Research Center, Moffett Field, California, USA

Since the launch of Landsat-1 28 years ago, remotely sensed data have been used to map features on the earth's surface. An increasing number of health studies have used remotely sensed data for monitoring, surveillance, or risk mapping, particularly of vector-borne diseases. Nearly all studies used data from Landsat, the French Système Pour l'Observation de la Terre, and the National Oceanic and Atmospheric Administration's Advanced Very High Resolution Radiometer. New sensor systems are in orbit, or soon to be launched, whose data may prove useful for characterizing and monitoring the spatial and temporal patterns of infectious diseases. Increased computing power and spatial modeling capabilities of geographic information systems could extend the use of remote sensing beyond the research community into operational disease surveillance and control. This article illustrates how remotely sensed data have been used in health applications and assesses earth-observing satellites that could detect and map environmental variables related to the distribution of vector-borne and other diseases.

Remote sensing data enable scientists to study the earth's biotic and abiotic components. These components and their changes have been mapped from space at several temporal and spatial scales since 1972. A small number of investigators in the health community have explored remotely sensed environmental factors that might be associated with disease-vector habitats and human transmission risk. However, most human health studies using remote sensing data have focused on data from Landsat's Multispectral Scanner (MSS) and Thematic Mapper (TM), the National Oceanic and Atmospheric Administration (NOAA)'s Advanced Very High Resolution Radiometer (AVHRR), and France's Système Pour l'Observation de la Terre (SPOT). In many of these studies (Table 1), remotely sensed data were used to derive three variables: vegetation cover, landscape structure, and water bodies.

International space agencies are planning an estimated 80 earth-observing missions in the next 15 years (29). During these missions >200 instruments will measure additional environmental features such as ocean color and other currently detectable variables, but at much higher spatial and spectral resolutions. The commercial sector is also planning to launch several systems in the next 5 years that could provide complementary data (30). These new capabilities will improve spectral, spatial, and temporal resolution, allowing exploration of risk factors previously beyond the capabilities of remote sensing. In addition, advances in pathogen, vector, and reservoir and host ecology have allowed assessment of a greater range of environmental factors that promote disease transmission, vector production, and the emergence and maintenance of disease foci, as well as risk for human-vector contact. Advances in computer processing and in geographic information system and global positioning system technologies facilitate integration of remotely sensed environmental parameters with health data so that models for disease surveillance and control can be developed.

In 1998, the National Aeronautics and Space Administration's (NASA) Center for Health Applications of Aerospace Related Technologies (CHAART)[1] evaluated current and planned

Address for correspondence: Louisa R. Beck, Earth Systems Science and Policy, California State University, Monterey Bay, MS 242-4, NASA Ames Research Center, Moffett Field, CA 94035-1000, USA; fax: 650-604-4680; e-mail: lrbeck@gaia.arc.nasa.gov.

[1]CHAART was established at Ames Research Center by NASA's Life Sciences Division, within the Office of Life & Microgravity Sciences & Applications, to make remote sensing, geographic information systems, global positioning systems, and computer modeling available to investigators in the human health community.

Table 1. Research using remote sensing data to map disease vectors[a]

| Disease | Vector | Location | Sensor | Ref. |
|---|---|---|---|---|
| Dracunculiasis | ***Cyclops*** spp. | Benin | TM | 1 |
| | ***Cyclops*** spp. | Nigeria | TM | 2 |
| Eastern equine encephalomyelitis | ***Culiseta melanura*** | Florida, USA | TM | 3 |
| Filariasis | ***Culex pipiens*** | Egypt | AVHRR | 4 |
| | ***Cx. pipiens*** | Egypt | TM | 5,6 |
| Leishmaniasis | ***Phlebotomus papatasi*** | SW Asia | AVHRR | 7 |
| Lyme disease | ***Ixodes scapularis*** | New York, USA | TM | 8,9 |
| | ***I. scapularis*** | Wisconsin, USA | TM | 10 |
| Malaria | ***Anopheles albimanus*** | Mexico | TM | 11 |
| | ***An. albimanus*** | Belize | SPOT | 12 |
| | ***An. albimanus*** | Belize | SPOT | 13 |
| | ***An. albimanus*** | Mexico | TM | 14 |
| | ***An.*** spp. | Gambia | AVHRR, Metosat | 15,16 |
| | ***An. albimanus*** | Mexico | TM | 17,18 |
| Rift Valley fever | ***Aedes*** & ***Cx***. spp. | Kenya | AVHRR | 19,20 |
| | ***Cx.*** spp. | Kenya | TM, SAR | 21 |
| | ***Cx.*** spp. | Senegal | SPOT, AVHRR | 22 |
| Schistosomiasis | ***Biomphalaria*** spp. | Egypt | AVHRR | 23 |
| Trypanosomiasis | ***Glossina*** spp. | Kenya, Uganda | AVHRR | 24 |
| | ***Glossina*** spp. | Kenya | TM | 25 |
| | ***Glossina*** spp. | West Africa | AVHRR | 26 |
| | ***Glossina*** spp. | Africa | AVHRR | 27 |
| | ***Glossina*** spp. | Southern Africa | AVHRR | 28 |

[a]See Appendix A for explanation of sensor acronyms satellite sensor systems as a first step in enabling human health scientists to determine data relevant for the epidemiologic, entomologic, and ecologic aspects of their research, as well as developing remote sensing-based models of transmission risk. This article discusses the results of the evaluation and presents two examples of how remotely sensed data have been used in health-related studies. The first example, a terrestrial application, illustrates how a single Landsat TM image was used to characterize the spatial patterns of key components of the Lyme disease transmission cycle in New York. The second example, which focuses on the coastal environment, shows how remote sensing data from different satellite systems can be combined to characterize and map environmental variables in the Bay of Bengal that are associated with the temporal patterns of cholera cases in Bangladesh. These examples demonstrate how remote sensing data acquired at various scales and spectral resolutions can be used to study infectious disease patterns.

### Lyme Disease in the Northeastern United States

During the past 10 years, NASA's Ames Research Center has been collaborating with the New York Medical College and the Yale School of Medicine to develop remote sensing-based models for mapping Lyme disease transmission risk in the northeastern United States (31,32). The first study compared Landsat TM data with canine seroprevalence rate (CSR) data summarized at the municipality level (31). The canine data were used as a measure of human exposure risk, the assumption being that dogs were more likely to acquire tick bites on or near their owner's property. The second study used TM data to map relative tick abundance on residential properties by using TM-derived indices of vegetation greenness and wetness (32). Figure 1 shows a subset of the TM data used in both studies, as well as some of the products (e.g., maps) derived from the data. Each product illustrated Lyme disease transmission variables, such as vector and reservoir habitats, as well as human risk for disease. Figure 1a shows raw Landsat-5 TM data, which are recorded in six spectral bands (excluding a seventh thermal band) at a spatial resolution of 30 m. These data were processed to derive the products shown in Figures 1b-d.

The image in Figure 1b was used to explore the relationship between forest patch size and deer distribution. Because white-tailed deer

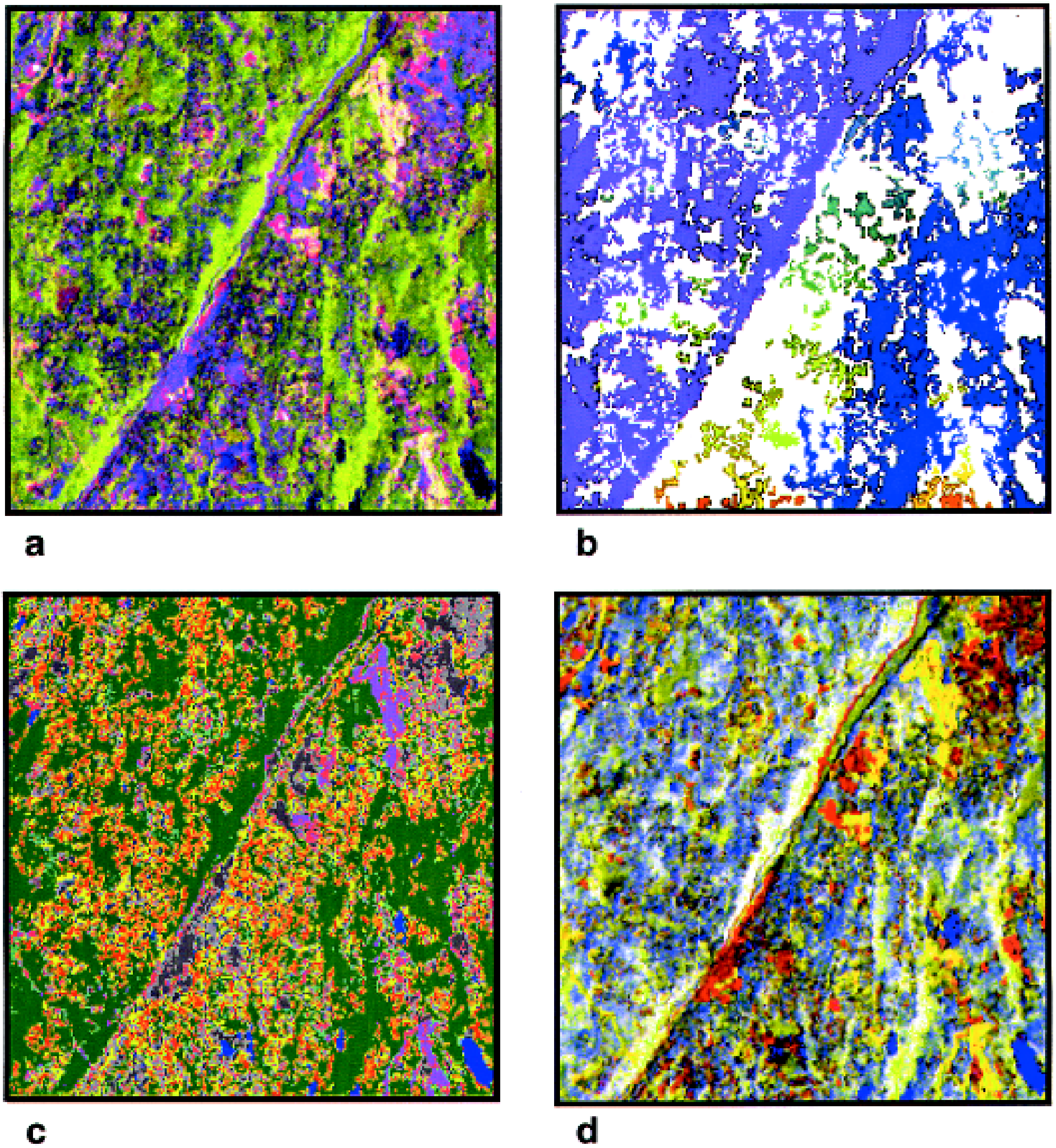

Figure 1. Landsat Thematic Mapper (TM) satellite data for a 6x6-km area in Westchester County, New York. Shown are the raw data (a), as well as products (e.g., maps) derived from the data (b-d) that might be used for modeling Lyme disease transmission risk. a) Raw Landsat TM image composed of bands 5, 4, and 3 (mid-infrared, near-infrared, and red bands). Vegetation is shown in shades of green, with bare soil and urban areas shown in shades of pink and purple. The spatial resolution of these data is 30x30 m. b) Map showing contiguous forest patches, derived from a Landsat TM classification. Colors represent discrete patches, with white indicating the absence of contiguous forest. c) A 12-class land cover map derived from the Landsat TM data. d) Composite image of three spectral indices derived from the Landsat TM data, showing the contributions of scene brightness in red, greenness in green, and wetness in blue.

serve as a major host of the adult tick as well as its primary mode of transportation, deer distribution was a potentially important factor in a Lyme disease risk model.

Figure 1c shows 12 classes used in two separate analyses of risk at two different scales (31). These classes include water, evergreen trees/vegetation, sparse deciduous trees, dense deciduous trees, clearings, golf courses (managed grass), urban/commercial, miscellaneous urban, residential-lawn, residential-sparse vegetation, residential-medium vegetation, and residential high-vegetation. In the first scale, the amount of remotely sensed deciduous forest was positively correlated (r-0.82) with canine exposure to ***Borrelia burgdorferi***, as indicated by CSR data summarized by municipality. In the second analysis, a linear regression of the residential-high vegetation pixels (i.e., wood-edge) and CSR data resulted in a correlation coefficient of 0.84—indicating that human-host contact risk (e.g., deer leaving the forest to feed on residential ornamental vegetation) might be a good measure of human-vector contact risk.

The image in Figure 1d was derived from the Landsat TM data by a Tasseled Cap Transformation (33). Tasseled Cap greenness and wetness were positively correlated with tick abundance on residential properties in this study area (32).

### Cholera in Bangladesh

The second example of the use of remotely sensed data to provide information for health research and applications concerns cholera in Bangladesh. In this study, described by Lobitz et al. (34), remotely sensed datasets, downloaded from the Internet at no cost, were used to search for temporal patterns in the Bay of Bengal associated with cholera outbreaks in Bangladesh.

Figure 2a shows a color-infrared image of the Ganges River, where it empties into the Bay of Bengal. These data, which were acquired by NOAA's AVHRR sensor, have a spatial resolution of 1.1 km. The sediment load, transported to the Bay of Bengal by the Ganges and Brahmaputra rivers, includes nutrients that could support plankton blooms. Plankton is an important marine reservoir of ***Vibrio cholerae***, which attaches primarily to zooplankton, which, in turn, is associated with phytoplankton (35).

In Figure 2b, the AVHRR data shown in Figure 2A were processed to show sea surface temperature (SST) (36). Because these data are for large-area studies, they have been processed at a spatial resolution of 18 km. Figure 2c represents sea surface height (SSH) anomaly data derived from the TOPEX/Poseidon satellite (37). These data have a spatial resolution of 1 degree. Increases in SST and SSH have preceded cholera outbreaks in Bangladesh (34).

In the next 15 years, new sensors will provide valuable data for studies of infectious diseases similar to the ones described here. For Lyme disease, new sensors could provide similar information about ecotones, human settlement patterns, or forests. These sensors include ARIES-1, scheduled for launch by Australia; CCD and IR/MSS sensors onboard CBERS, launched by China and Brazil in late 1999; Ikonos, a commercial satellite with 4-m spatial resolution; LISS III, onboard the orbiting Indian IRS-1C and –1D satellites; and ASTER, onboard the recently launched Terra satellite. Information from these sensors could also be used to address other vector-borne diseases, such as malaria, schistosomiasis, trypanosomiasis, and hantavirus, whose patterns are likewise influenced by environmental variables.

SeaWiFS, the Sea-Viewing Wide Field-of-View Sensor, with its increased spectral resolution of 1.1 km, is already providing imagery critical to understanding the temporal and spatial pattern of cholera risk (35). This sensor was specifically designed to gather information about ocean color (38) (Figure 2d).

### Sensor Evaluation Project[2]

CHAART evaluated data from current and planned satellite instruments for mapping, surveillance, prediction, and control of human disease transmission activities, including vector ecology, reservoir and host ecology, and human settlement patterns. From hundreds of potential sensors, 54 were identified that were current (or would be launched within the next 5 years), operational (not reserved for the scientific community), and digital (not photographic).

Beginning in 1985, NASA has held a series of workshops to elicit input from the health community on the use of remote sensing in the areas of entomology, ecology, epidemiology, vector control, and infectious diseases. In addition, NASA has participated in sessions on remote sensing and health at professional meetings sponsored by national and international health

[2]The information gathered during the CHAART sensor evaluation process is available at http://geo.arc.nasa.gov/sge/health/sensor/sensor.html.

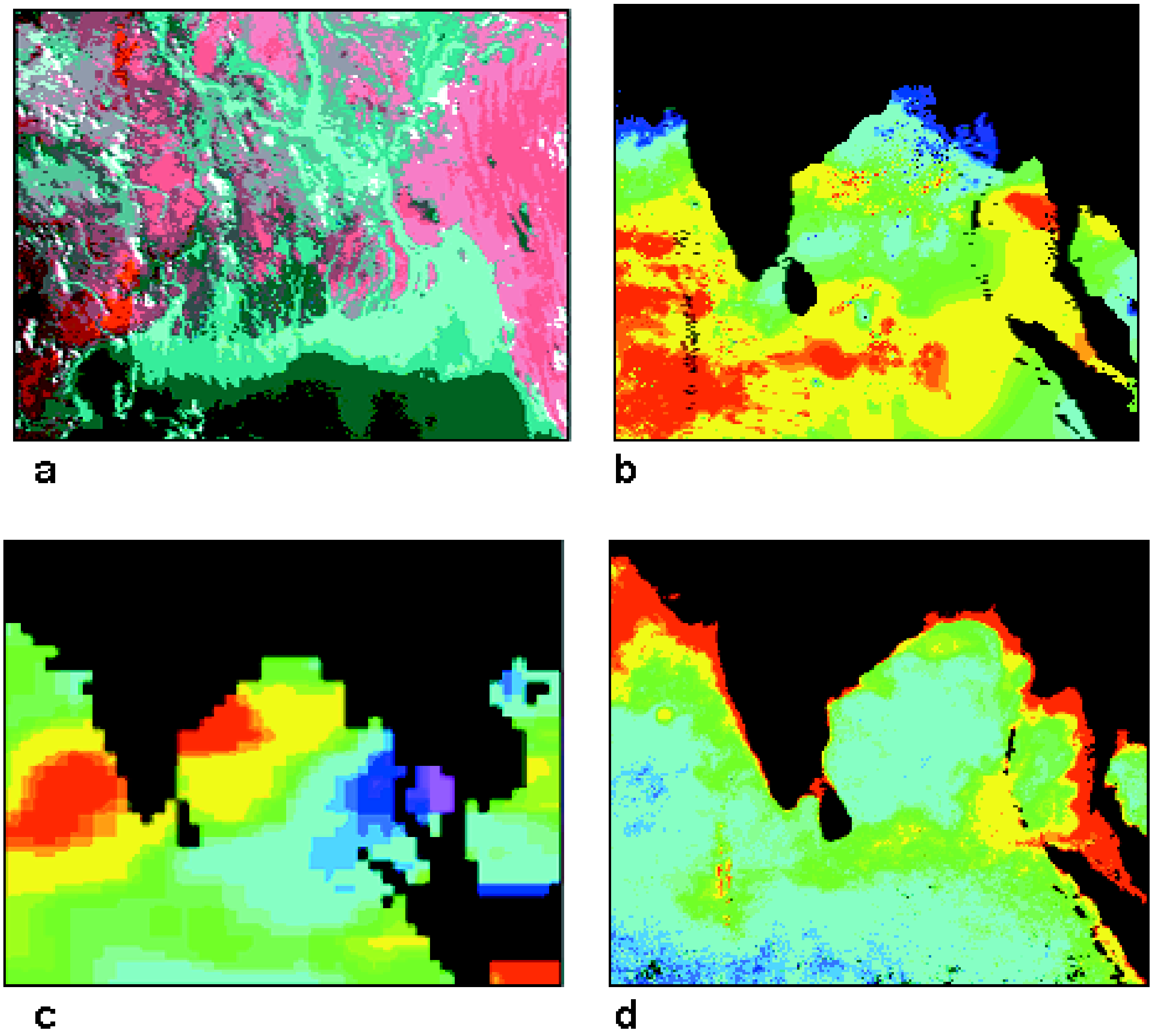

Figure 2. Datasets used to model the temporal patterns of cholera outbreaks in Bangladesh. a) Advanced Very High Resolution Radiometer (AVHRR) satellite image showing the mouth of the Ganges River and the Bay of Bengal. Vegetation is shown in shades of red and water in shades of blue. The spatial resolution of these data is 1.1 km. b) Sea surface temperature data, derived from AVHRR thermal bands. Temperatures range from low (purple) to high (red). c) Sea surface height data, derived from TOPEX/Poseidon satellite data. The spatial resolution of these data is 1 degree. d) Image derived from the Sea-Viewing Wide Field-of-View Sensor (SeaWiFS) showing chlorophyll concentration, ranging from low (blue) to high (red). These satellite data have a nominal spatial resolution of 1.1 km.

organizations. On the basis of this experience as well as a review of the scientific literature (Table 1), there does not appear to be consensus in the health community regarding requirements for a remote sensing system. Some investigators use remotely sensed data to resolve questions regarding the relationship between an aspect of disease transmission and an environmental variable. Other researchers already have a model of disease transmission and have specific spatial, temporal, or spectral requirements for the remotely sensed variables.

No single spatial, temporal, or spectral resolution is universally appropriate for understanding the transmission risk for any disease, given the variety of vectors, reservoirs, hosts, geographic locations, and environmental variables associated with that disease. Therefore, in evaluating the existing sensors, CHAART used an approach that allowed individual investigators to identify satellite data appropriate for their own needs. This approach defined 16 groups of physical factors that could be used for both research and applications. Each factor is essentially an environmental variable that might have a direct or indirect bearing on the survival of pathogens, vectors, reservoirs, and hosts. These factors may also affect many types of non-vector-borne diseases, such as waterborne diseases. The factors are vegetation or crop type, vegetation green-up, ecotones, deforestation, forest patches, flooded forests, general flooding, permanent water, wetlands, soil moisture, canals, human settlements, urban features, ocean color, SST, and SSH. Precipitation, humidity, and surface temperature were not included because deriving these measurements from raw data requires highly specialized processing and calibration, routinely performed by qualified groups who often make the information available on Internet websites (Appendix B).

The sensor evaluation project generated a series of tables that associated each of the 16 factors with the 54 sensors according to spatial, temporal, and spectral characteristics. For example, factors requiring frequent monitoring, such as vegetation green-up, are linked with sensors with shorter repeat overpasses. Similarly, factors requiring very high spatial resolution, such as mapping urban features, are linked with sensors having a spatial resolution of 10 m or less, regardless of their temporal or spectral resolutions.

Perhaps the broadest use of Landsat and SPOT data has been to identify and map vegetation or crop types. This factor is important because the distribution of vegetation types integrates the combined impact of rainfall, temperature, humidity, topographic effects, soil, water availability, and human activities. Nearly all vector-borne diseases are linked to the vegetated environment during some aspect of their transmission cycle; in many cases, this vegetation can be sensed remotely from space. The spatial and temporal distribution of vector or reservoir and host species may relate to the occurrence and distribution of specific vegetation or crop types, not simply to whether an area has forests or grasslands. For example, food and cover preferences of the white-tailed deer, the host for adult ticks that transmit Lyme disease in the northeastern United States, might well encourage deer to live near certain types of forest. Crop-type information may also be important for studying the effects of pesticides (e.g., vector resistance; illnesses caused by exposure to toxins).

The sensor evaluation procedure has identified many potentially useful sensors for mapping vegetation and crop type beyond the Landsat and SPOT systems (Table 2). A ground resolution threshold of 30 m was used as the upper limit for exploring the relationship between vegetation (or crop) type and disease vectors, reservoirs, and hosts; above 30 m, vegetation and crop type are more difficult to ascertain. Many of the sensors could also be used for mapping the boundary

Table 2. Current and proposed sensor systems for identifying and mapping vegetation and crop type[a]

| Temporal | Spatial resolution[b] (m) | | |
|---|---|---|---|
| resolution (d) | 1-5 | 6-10 | 11-30 |
| Daily | (QuickBird)[c] | | |
| 2-7 | (Orbview-3,4)<br>(QuickBird) | (Almaz-1b MSU-E2)<br>(ALOS AVNIR-2)<br>(Orbview-4)<br>(SPOT-5a,b 3xHRG) | (ALOS AVNIR-2)<br>(ARIES-1)<br>SPOT-4 2xHRVIR |
| 8-14 | Ikonos | Priroda/Mir MOMS-2P | Priroda/Mir MOMS-2P |
| 15-30 | | | Terra ASTER<br>IRS-1C,D LISS III<br>Landsat TM<br>Landsat-7 ETM+<br>SPOT-2 2xHRV |
| >30 | | (ALOS AVNIR-2) | (ALOS AVNIR-2) |

[a]This matrix is the output from an interactive search with the search engine located at http://geo.arc.nasa.gov/sge/health/sensor/senchar.html.
[b]See Appendix A for explanations of sensor acronyms.
[c]Sensors in parentheses have not yet been launched.

between vegetation types, or ecotones, which provide habitat for insects and animals critical to the maintenance and transmission of vector-borne diseases. These edges may be areas for increased risk for vector-human contact, as indicated by the relationship between Lyme disease transmission and suburban encroachment into forested areas in the northeastern United States. The movement of humans into forested edges where potential vectors are established could also be important for predicting malaria or yellow fever transmission.

The list of 16 factors used in the CHAART evaluation includes some that have not yet been quantified because available sensors do not provide adequate spatial, spectral, or temporal resolutions. Two of these factors are briefly described below to illustrate how remotely sensed data might be used to explore their potential links to human health. More links between the factors and various diseases are listed in Table 3.

Table 3. Potential links between remotely sensed factors and disease

| Factor | Disease | Mapping opportunity |
|---|---|---|
| Vegetation/crop type | Chagas disease | Palm forest, dry & degraded woodland habitat for triatomines |
| | Hantavirus | Preferred food sources for host/reservoirs |
| | Leishmaniasis | Thick forests as vector/reservoir habitat in Americas |
| | Lyme disease | Preferred food sources and habitat for host/reservoirs |
| | Malaria | Breeding/resting/feeding habitats; crop pesticides ➔ vector resistance |
| | Plague | Prairie dog and other reservoir habitat |
| | Schistosomiasis | Agricultural association with snails, use of human fertilizer |
| | Trypanosomiasis | ***Glossina*** habitat (forests, around villages, depending on species) |
| | Yellow fever | Reservoir (monkey) habitat |
| Vegetation green-up | Hantavirus | Timing of food sources for rodent reservoirs |
| | Lyme disease | Habitat formation and movement of reservoirs, hosts, vectors |
| | Malaria | Timing of habitat creation |
| | Plague | Locating prairie dog towns |
| | Rift Valley fever | Rainfall |
| | Trypanosomiasis | ***Glossina*** survival |
| Ecotones | Leishmaniasis | Habitats in and around cities that support reservoir (e.g., foxes) |
| | Lyme disease | Ecotonal habitat for deer, other hosts/reservoirs; human/vector contact risk. |
| Deforestation | Chagas disease | New settlements in endemic-disease areas |
| | Malaria | Habitat creation (for vectors requiring sunlit pools) |
| | | Habitat destruction (for vectors requiring shaded pools) |
| | Yellow fever | Migration of infected workers into forests where vectors exist |
| | | Migration of disease reservoirs (monkeys) in search of new habitat |
| Forest patches | Lyme disease | Habitat requirements of deer and other hosts, reservoirs |
| | Yellow fever | Reservoir (monkey) habitat, migration routes |
| Flooded forests | Malaria | Mosquito habitat |
| Flooding | Malaria | Mosquito habitat |
| | Rift Valley fever | Flooding of dambos, breeding habitat for mosquito vector |
| | Schistosomiasis | Habitat creation for snails |
| | St. Louis encephalitis | Habitat creation for mosquitoes |
| Permanent water | Filariasis | Breeding habitat for ***Mansonia*** mosquitoes |
| | Malaria | Breeding habitat for mosquitoes |
| | Onchocerciasis | ***Simulium*** larval habitat |
| | Schistosomiasis | Snail habitat |
| Wetlands | Cholera | ***Vibrio cholerae*** associated with inland water |
| | Encephalitis | Mosquito habitat |
| | Malaria | Mosquito habitat |
| | Schistosomiasis | Snail habitat |
| Soil moisture | Helminthiases | Worm habitat |
| | Lyme disease | Tick habitat |
| | Malaria | Vector breeding habitat |
| | Schistosomiasis | Snail habitat |
| Canals | Malaria | Dry season mosquito-breeding habitat; ponding; leaking water |
| | Onchocerciasis | ***Simulium*** larval habitat |
| | Schistosomiasis | Snail habitat |
| Human settlements | Diseases | Source of infected humans; populations at risk for transmission in general |
| Urban features | Chagas disease | Dwellings that provide habitat for triatomines |
| | Dengue fever | Urban mosquito habitats |
| | Filariasis | Urban mosquito habitats |
| | Leishmaniasis | Housing quality |
| Ocean color (Red tides) | Cholera | Phytoplankton blooms; nutrients, sediments |
| Sea surface temp. | Cholera | Plankton blooms (cold water upwelling in marine environment) |
| Sea surface height | Cholera | Inland movement of ***Vibrio***-contaminated tidal water |

### Urban Features

The detection of urban features requires higher spatial resolution systems than needed for detecting the presence of human settlements. Some disease vectors are associated with specific urban features such as housing type, which can only be detected by sensors with very high spatial resolution. In the future, new sensors may be able to provide information on the urban environment (Table 4).

Table 4. Current and proposed sensor systems for identifying and mapping urban features[a]

| Temporal resolution (d) | Spatial resolution[b] (m) 1-5 | 6-10 |
|---|---|---|
| Daily | (QuickBird)[c] | |
| 2-7 | (ALOS AVNIR-2)<br>(Orbview-3,4)<br>(QuickBird)<br>(SPOT-5a,b 3xHRVIR) | (Almaz-1b MSU-E2)<br>(ALOS AVNIR-2)<br>(ARIES-1)<br>IRS-1C,D PAN<br>(Orbview-4)<br>SPOT-4 2xHRVIR<br>(SPOT-5a,b 3xHRVIR) |
| 8-15 | Ikonos | IRS-1C,D PAN<br>Priroda/Mir MOMS-2P |
| 15-30 | | IRS-1C,D PAN<br>SPOT-2 2xHRV |
| >30 | (ALOS AVNIR-2) | (ALOS AVNIR-2) |

[a]This matrix is the output from an interactive search with the search engine located at http://geo.arc.nasa.gov/sge/health/sensor/senchar.html

[b]See Appendix A for explanations of sensor acronyms.

[c]Sensors in parentheses have not yet been launched.

### Soil Moisture

Wet soils indicate a suitable habitat for species of snails, mosquito larvae, ticks, and worms. Several types of sensors can detect soil moisture, including synthetic aperture radars (SARs), shortwave-infrared, and thermal-infrared sensors (Table 5). SARs are particularly important for sensing ground conditions in areas of cloud cover or vegetation canopy cover, two factors commonly found in the tropics.

### Conclusions

The extent to which remotely sensed data are used for studying the spatial and temporal patterns of disease depends on a number of obstacles and opportunities. Many of the obstacles—including cost, inadequate spatial, spectral, or temporal resolutions, and long turnaround times for products—have restricted the use of remote sensing within the user community as a whole. Many of these barriers will be addressed by new sensor systems in the next 5 years. The recently launched Landsat-7 ETM+ sensor, for example, is now providing 30-m multispectral data, a 15-m panchromatic band, and an improved 60-m thermal infrared band, all at a cost that is an order of magnitude less than current Landsat-5 TM data.

With the higher spatial and spectral resolutions, more frequent coverage, lower price,

Table 5. Current and proposed sensor systems for identifying and mapping soil moisture[a]

| Temporal resolution (d) | Spatial Resolution[b] (m) 11-30 | 101-500 | 501-1,000 | 1,001-4,000 |
|---|---|---|---|---|
| Daily | | | | NOAA AVHRR |
| 2-7 | (Almaz-1b SAR-70)[c]<br>(ARIES-1)<br>(ENVISAT-1 ASAR)<br>Radarsat<br>SPOT-4 2xHRVIR | | (ADEOS II GLI)<br>(Almaz-1b MSU-SK)<br>(Almaz-1b SROSM)<br>(ENVISAT-1 AATSR)<br>Terra MODIS<br>(EOS PM-1 MODIS)<br>Resurs-01 N2,3<br>MSU-SK | |
| 8-14 | (LightSAR)<br>Priroda/Mir MOMS-2P | | Priroda/Mir MSU-SK | |
| 15-30 | Terra ASTER<br>ERS-1,2 AMI-SAR<br>Landsat TM<br>Landsat-7 ETM+ | Landsat TM TIR | | |
| >30 | ERS-1,2 AMI-SAR | | | |

[a]This matrix is the output from an interactive search with the search engine located at http://geo.arc.nasa.gov/sge/health/sensor/senchar.html.

[b]See Appendix A for explanations of sensor acronyms.

[c]Sensors in parentheses have not yet been launched.

and increased availability offered by the range of new sensors, human health investigators should be able to extract many more environmental variables than previously realized. These improvements will provide new opportunities to extend the uses of remote sensing technology beyond a few vector-borne diseases to studies of water- and soil-borne diseases (for example, cholera and schistosomiasis [waterborne] and the helminthiases) and the mapping of human settlements at risk. The next generation of earth-observing remote sensing systems will also allow investigators in the human health community to characterize an increasing range of variables key to understanding the spatial and temporal patterns of disease transmission risk. These improved capabilities, when combined with the increased computing power and spatial modeling capabilities of geographic information systems, should extend remote sensing into operational disease surveillance and control.

### Acknowledgment

The CHAART staff acknowledges the contributions made to the sensor project by their colleague Michael A. Spanner (1952-1998).

Funding for the sensor evaluation project was provided by the Office of Earth Science at NASA Headquarters, RW#179. Funding for the Lyme disease and cholera examples described in this article was provided by NASA's Life Sciences Division and the NASA Ames Director's Discretionary Fund.

### References

1. Clarke KC, Osleeb JR, Sherry JM, Meert JP, Larsson RW. The use of remote sensing and geographic information systems in UNICEF's dracunculiasis (Guinea worm) eradication effort. Prev Vet Med 1990;11:229-35.
2. Ahearn SC, De Rooy C. Monitoring the effects of dracunculiasis remediation on agricultural productivity using satellite data. International Journal of Remote Sensing 1996;17:917-29.
3. Freier JW. Eastern equine encephalomyelitis. Lancet 1993;342:1281-3.
4. Thompson DF, Malone JB, Harb M, Faris R, Huh OK, Buck AA, et al. Bancroftian filariasis distribution and diurnal temperature differences in the southern Nile Delta. Emerg Infect Dis 1996;2:234-5.
5. Hassan AN, Beck LR, Dister S. Prediction of villages at risk for filariasis transmission in the Nile Delta using remote sensing and geographic information system technologies. J Egypt Soc Parasitol 1998;28:75-87.
6. Hassan AN, Dister S, Beck L. Spatial analysis of lymphatic filariasis distribution in the Nile Delta in relation to some environmental variables using geographic information system technology. J Egypt Soc Parasitol 1998;28:119-31.
7. Cross ER, Newcomb WW, Tucker CJ. Use of weather data and remote sensing to predict the geographic and seasonal distribution of ***Phlebotomus paptasi*** in Southwest Asia. Am J Trop Med Hyg 1996;54:530-6.
8. Dister SW, Beck LR, Wood BL, Falco R, Fish D. The use of GIS and remote sensing technologies in a landscape approach to the study of Lyme disease transmission risk. Proceedings of GIS '93: Geographic Information Systems in Forestry, Environmental and Natural Resource Management; 1993 Feb 15-18; Vancouver, B.C., Canada. 1993.
9. Dister SW, Fish D, Bros S, Frank DH, Wood BL. Landscape characterization of peridomestic risk for Lyme disease using satellite imagery. Am J Trop Med Hyg 1997;57:687-92.
10. Kitron U, Kazmierczak JJ. Spatial analysis of the distribution of Lyme disease in Wisconsin. Am J Epidemiol 1997;145:558-66.
11. Pope KO, Rejmánková E, Savage HM, Arredondo-Jimenez JI, Rodríguez MH, Roberts DR. Remote sensing of tropical wetlands for malaria control in Chiapas, Mexico. Ecological Applications 1993;4:81-90.
12. Rejmánková E, Roberts DR, Pawley A, Manguin S, Polanco J. Predictions of adult ***Anopheles albimanus*** densities in villages based on distance to remotely sensed larval habitats. Am J Trop Meg Hyg 1995;53 (5):482-488.
13. Roberts DR, Paris JF, Manguin S, Harbach RE, Woodruff R, Rejmánková E, et al. Predictions of malaria vector distribution in Belize based on multispectral satellite data. Am J Trop Med Hyg 1996;54:304-8.
14. Rodríguez AD, Rodríguez MH, Hernández JE, Dister SW, Beck LR, Rejmánková E, et al. Landscape surrounding human settlements and malaria mosquito abundance in southern Chiapas, Mexico. J Med Entomol 1996;33:39-48.
15. Thomson MC, Connor SJ, Milligan PJM, Flasse SP. The ecology of malaria-as seen from Earth-observation satellites. Ann Trop Med Parasitol 1996;90:243-64.
16. Thomson MC, Connor SJ, Milligan PJM, Flasse SP. Mapping malaria risk in Africa: what can satellite data contribute? Parsitology Today 1997;13:313-8.
17. Beck LR, Rodríguez MH, Dister SW, Rodríguez AD, Rejmánková E, Ulloa A, et al. Remote sensing as a landscape epidemiologic tool to identify villages at high risk for malaria transmission. Am J Trop Med Hyg 1994;51:271-80.
18. Beck LR, Rodríguez MH, Dister SW, Rodríguez AD, Washino RK, Roberts DR, et al. Assessment of a remote sensing based model for predicting malaria transmission risk in villages of Chiapas, Mexico. Am J Trop Med Hyg 1997;56:99-106.
19. Linthicum KJ, Bailey CL, Davies FG, Tucker CJ. Detection of Rift Valley Fever viral activity in Kenya by satellite remote sensing imagery. Science 1987;235:1656-9.
20. Linthicum KJ, Bailey CL, Tucker CJ, Mitchell KD, Logan TM, Davies FG, et al. Applications of polar-orbiting, meteorological satellite data to detect flooding in Rift Valley Fever virus vector mosquito habitats in Kenya. Med Vet Entomol 1990;4:433-8.

21. Pope KO, Sheffner EJ, Linthicum KJ, Bailey CL, Logan TM, Kasischke ES, et al. Identification of central Kenyan Rift Valley Fever virus vector habitats with Landsat TM and evaluation of their flooding status with Airborne Imaging Radar. Remote Sensing Environment 1992;40:185-96.
22. Linthicum KJ, Bailey CL, Tucker CJ, Gordon SW, Logan TM, Peters CJ, et al. Man-made ecological alterations of Senegal River basin on Rift Valley Fever transmission. Sistema Terra 1994;45-7.
23. Malone JB, Huh OK, Fehler DP, Wilson PA, Wilensky DE, Holmes RA, et al. Temperature data from satellite images and the distribution of schistosomiasis in Egypt. Am J Trop Med Hyg 1994;50:714-22.
24. Rogers DJ. Satellite imagery, tsetse and trypanosomiasis. Prev Vet Med 1991;11:201-20.
25. Kitron U, Otieno LH, Hungerford LL, Odulaja A, Brigham WU, Okello OO, et al. Spatial analysis of the distribution of tsetse flies in the Lambwe Valley, Kenya, using Landsat TM satellite imagery and GIS. Journal of Animal Ecology 1996;65:371-80.
26. Rogers DJ, Randolph SE. Mortality rates and population density of tsetse flies correlated with satellite imagery. Nature 1991;351:739-41.
27. Rogers DJ, Williams BG. Monitoring trypanosomiasis in space and time. Parasitology 1993;106 Suppl:77-92.
28. Robinson TP, Rogers DJ, Williams B. Mapping tsetse habitat suitability in the common fly belt of Southern Africa using multivariate analysis of climate and remotely sensed data. Med Vet Entomol 1997;11:235-45.
29. Committee on Earth Observation Satellites. Coordination for the Next Decade (1995 CEOS Yearbook). European Space Agency. Smith System Engineering Ltd, UK; 1995.
30. Stoney WE. The Pecora legacy-land observation satellites in the next century. Proceedings of the Pecora 13 Symposium; 1996 Aug 20-22; Sioux Falls, SD; Bethesda, MD: American Society for Photogrammetry and Remote Sensing; 1998. p. 260-74.
31. Dister SW, Beck LR, Wood BL, Falco R, Fish D. The use of GIS and remote sensing technologies in a landscape approach to the study of Lyme disease transmission risk. In: Proceedings of GIS '93: Geographic Information Systems in Forestry, Environmental and Natural Resource Management. Vancouver, B.C., Canada; 1993.
32. Dister SW, Fish D, Bros S, Frank DH, Wood BL. Landscape characterization of peridomestic risk for Lyme disease using satellite imagery. Am J Trop Med Hyg 1997;57:687-92.
33. Crist EP, Cicone RC. A physically based transformation of Thematic Mapper data—The TM Tasseled Cap. IEEE Trans Geosciences and Remote Sensing 1984;22:256-63.
34. Lobitz B, Beck L, Huq A, Wood B, Fuchs G, Faroque ASG, et al. Climate and infectious disease: use of remote sensing for detection of ***Vibrio cholerae*** by indirect measurement. Proc National Academy Sciences 2000;97:1438-43.
35. Huq A, Colwell RR. Vibrios in the marine and estuarine environments. J Marine Biotechnology 1995;3:60-3.
36. NASA Jet Propulsion Laboratory, Physical Oceanography Distributed Active Archive Center. Pasadena, California; 1996. Archived data available at the following URL: http://podaac.jpl.nasa.gov
37. Center for Space Research. University of Texas, Austin; 1996. Archived data available at the following URL: http://www.csr.utexas.edu
38. NASA Goddard Distributed Active Archive Center, Greenbelt, Maryland; 1996. Archived data available at the following URL: http://seawifs.gsfc.nasa.gov/cgibrs/level3.pl

Appendix A. Acronyms used in the text and tables

| Acronym | Mission | Instruments | Country |
|---|---|---|---|
| ADEOS II | Advanced Earth Observation Satellite | GLI | Japan |
| ALOS | Advanced Land Observing Satellite | AVNIR | Japan |
| ARIES | Australian Resource Information & Environment Satellite | ARIES | Australia |
| CBERS | China-Brazil Earth Resources Satellite | CCD, IR/MSS | China/Brazil |
| ENVISAT | Environmental Satellite | AATSR, ASAR | Europe |
| EOS | Earth Observation System | ASTER, MODIS | USA |
| ERS-2 | ESA (European Space Agency) Remote Sensing | AMI-SAR | Europe |
| IRS | Indian Remote Sensing Satellite | PAN, LISS | India |
| NOAA | National Oceanographic & Atmospheric Administration | AVHRR | USA |
| SPOT | Système Pour l'Observation de la Terre | HRV, HRVIR | France |
| **Acronym** | **Instrument** | **Mission** | **Country** |
| AATSR | Advanced Along Track Scanning Radiometer | ENVISAT 1 | ESA |
| AMI-SAR | Active Microwave Instrumentation Synthetic Aperture Radar | ERS-1, 2 | ESA |
| ASAR | Advanced Synthetic Aperture Radar | ENVISAT 1 | ESA |
| ASTER | Advanced Spaceborne Thermal Emission & Reflection Radiometer | Terra | USA |
| AVHRR | Advanced Very High Resolution Radiometer | NOAA | USA |
| AVNIR | Advanced Visible & Near Infrared Radiometer | ALOS | Japan |
| CCD | Charged Couple Device Camera | CBERS | China/Brazil |
| ETM+ | Enhanced Thematic Mapper Plus | Landsat-7 | USA |
| GLI | Global Land Imager | ADEOS II | Japan |
| HRV | High Resolution Visible | SPOT 1, 2 | France |
| HRVIR | High Resolution Visible & Infrared | SPOT 4, 5 | France |
| IR-MSS | Infrared-Multispectral Scanner | CBERS | China/Brazil |
| LISS III | Linear Imaging Self-Scanning System | IRS-1C, D | India |
| MODIS | Moderate Resolution Imaging Spectro Radiometer | Terra, EOS PM 1-3 | USA |
| MOMS-2P | Modular Optoelectronic Multispectral Scanner | Priroda/Mir | Russia |
| MSU-E2 | Multizone High-Resolution Electronic Scanner | Almaz-1B | Russia |
| MSU-SK | Multizone Middle-Resolution Optomechanical Scanner | Almaz-1B | Russia |
| | | Priroda | Russia |
| | | Resurs-O1, O2 | Russia |
| PAN | Panchromatic | IRS-1C, D | India |
| PAN | Panchromatic | Ikonos-2 | Space Imaging |
| SAR-70 | Synthetic Aperture Radar (70 cm) | Almaz-1B | Russia |
| SeaWiFS | Sea-Viewing Wide Field-of-View Sensor | TOPEX/Poseidon | France/USA |
| SROSM | Spectroradiometer for Ocean Satellite Monitoring | Almaz-1B | Russia |
| TM | Thematic Mapper | Landsat | USA |
| **Acronym** | **Miscellaneous** | | |
| ESA | European Space Agency | | |
| TIR | Thermal Infrared | | |

Appendix B. Partial list of Internet locations that contain (or will contain) products derived from remotely sensed precipitation, moisture, relative humidity, and surface temperature data

| Parameter | Mission/ sensor[a] | Spatial | Temporal[b] | Web site |
|---|---|---|---|---|
| Precipitation | TRMM/TMI | 10 km | D | daac.gsfc.nasa.gov/CAMPAIGN_DOCS/hydrology/hd_main.html |
| Precipitation | TRMM/TMI | 5° | M | daac.gsfc.nasa.gov/CAMPAIGN_DOCS/hydrology/hd_main.html |
| Precipitation | Terra[c]/DAS[d] | 1° | 8-day | eos-am.gsfc.nasa.gov |
| Precipitation | GOES/Sounder | 10 km | hr | www.nndc.noaa.gov/phase3/productaccm.htm |
| Precipitation rate | Terra[c]/DAS[d] | 2° | 8/day | eos-am.gsfc.nasa.gov |
| Precipitation amount | TOVS/MSU | 0.5° | D, M, Y | ghrc.msfc.nasa.gov/ghrc/list.html |
| Moisture | GOES/Imager | 8 km | hr | www.nndc.noaa.gov/phase3/productaccm.htm |
| Relative humidity | Terra[c]/DAS[d] | 2° | 4/day | eos-am.gsfc.nasa.gov |
| Surface temperature | Terra[c]/MODIS | 1 km | D | eos-am.gsfc.nasa.gov |
| Surface temperature | Terra[c]/MODIS | 1° | D, 8-day, M | eos-am.gsfc.nasa.gov |
| Surface temperature | Terra[c]/DAS[d] | 2° | 8/day | eos-am.gsfc.nasa.gov |
| Surface temperature | Envisat/AATSR | 17 km | D | envisat.estec.esa.nl/envisat-welcom.html |
| Surface temperature | Envisat/AATSR | 50 km | D | envisat.estec.esa.nl/envisat-welcom.html |
| Surface temperature | ERS/ATSR | 1 km | W | earth1.esrin.esa.it/ERS |
| Surface temperature | Meteosat/VISSR | 5 km | 48/day | www.eumetsat.de/en |
| Surface temperature | GOES/Imager | 4 km | hr | www.nndc.noaa.gov/phase3/productaccm.htm |

[a]See Appendix A for explanation of sensor acronyms.
[b]hr, hourly; D, daily; W, weekly; M, monthly; Y, yearly.
[c]Future launch.
[d]Data Assimilation System.

## References

[PDF_00664] Beck L. R., Rodriguez M. H., Dister S. W., Rodriguez A. D., Rejmankova E., Ulloa A., Meza R. A., Roberts D. R., Paris J. F., Spanner M. A. (1994). Remote sensing as a landscape epidemiologic tool to identify villages at high risk for malaria transmission.. Am J Trop Med Hyg.

[PDF_00669] Beck L. R., Rodriguez M. H., Dister S. W., Rodriguez A. D., Washino R. K., Roberts D. R., Spanner M. A. (1997). Assessment of a remote sensing-based model for predicting malaria transmission risk in villages of Chiapas, Mexico.. Am J Trop Med Hyg.

[PDF_00621] Cross E. R., Newcomb W. W., Tucker C. J. (1996). Use of weather data and remote sensing to predict the geographic and seasonal distribution of Phlebotomus papatasi in southwest Asia.. Am J Trop Med Hyg.

[PDF_00632] Dister S. W., Fish D., Bros S. M., Frank D. H., Wood B. L. (1997). Landscape characterization of peridomestic risk for Lyme disease using satellite imagery.. Am J Trop Med Hyg.

[PDF_00730] Dister S. W., Fish D., Bros S. M., Frank D. H., Wood B. L. (1997). Landscape characterization of peridomestic risk for Lyme disease using satellite imagery.. Am J Trop Med Hyg.

[PDF_00606] Freier J. E. (1993). Eastern equine encephalomyelitis.. Lancet.

[PDF_00612] Hassan A. N., Beck L. R., Dister S. (1998). Prediction of villages at risk for filariasis transmission in the Nile Delta using remote sensing and geographic information system technologies.. J Egypt Soc Parasitol.

[PDF_00616] Hassan A. N., Dister S., Beck L. (1998). Spatial analysis of lymphatic filariasis distribution in the Nile Delta in relation to some environmental variables using geographic information system technology.. J Egypt Soc Parasitol.

[PDF_00636] Kitron U., Kazmierczak J. J. (1997). Spatial analysis of the distribution of Lyme disease in Wisconsin.. Am J Epidemiol.

[PDF_00674] Linthicum K. J., Bailey C. L., Davies F. G., Tucker C. J. (1987). Detection of Rift Valley fever viral activity in Kenya by satellite remote sensing imagery.. Science.

[PDF_00678] Linthicum K. J., Bailey C. L., Tucker C. J., Mitchell K. D., Logan T. M., Davies F. G., Kamau C. W., Thande P. C., Wagateh J. N. (1990). Application of polar-orbiting, meteorological satellite data to detect flooding of Rift Valley Fever virus vector mosquito habitats in Kenya.. Med Vet Entomol.

[PDF_00738] Lobitz B., Beck L., Huq A., Wood B., Fuchs G., Faruque A. S., Colwell R. (2000). Climate and infectious disease: use of remote sensing for detection of Vibrio cholerae by indirect measurement.. Proc Natl Acad Sci U S A.

[PDF_00695] Malone J. B., Huh O. K., Fehler D. P., Wilson P. A., Wilensky D. E., Holmes R. A., Elmagdoub A. I. (1994). Temperature data from satellite imagery and the distribution of schistosomiasis in Egypt.. Am J Trop Med Hyg.

[PDF_00639] Pope K. O., Rejmankova E., Savage H. M., Arredondo-Jimenez J. I., Rodriguez M. H., Roberts D. R. (1994). Remote sensing of tropical wetlands for malaria control in Chiapas, Mexico.. Ecol Appl.

[PDF_00643] Rejmankova E., Roberts D. R., Pawley A., Manguin S., Polanco J. (1995). Predictions of adult Anopheles albimanus densities in villages based on distances to remotely sensed larval habitats.. Am J Trop Med Hyg.

[PDF_00648] Roberts D. R., Paris J. F., Manguin S., Harbach R. E., Woodruff R., Rejmankova E., Polanco J., Wullschleger B., Legters L. J. (1996). Predictions of malaria vector distribution in Belize based on multispectral satellite data.. Am J Trop Med Hyg.

[PDF_00711] Robinson T., Rogers D., Williams B. (1997). Mapping tsetse habitat suitability in the common fly belt of southern Africa using multivariate analysis of climate and remotely sensed vegetation data.. Med Vet Entomol.

[PDF_00653] Rodriguez A. D., Rodriguez M. H., Hernandez J. E., Dister S. W., Beck L. R., Rejmankova E., Roberts D. R. (1996). Landscape surrounding human settlements and Anopheles albimanus (Diptera: Culicidae) abundance in Southern Chiapas, Mexico.. J Med Entomol.

[PDF_00706] Rogers D. J., Randolph S. E. (1991). Mortality rates and population density of tsetse flies correlated with satellite imagery.. Nature.

[PDF_00608] Thompson D. F., Malone J. B., Harb M., Faris R., Huh O. K., Buck A. A., Cline B. L. (1996). Bancroftian filariasis distribution and diurnal temperature differences in the southern Nile delta.. Emerg Infect Dis.

[PDF_00658] Thomson M. C., Connor S. J., Milligan P. J., Flasse S. P. (1996). The ecology of malaria--as seen from Earth-observation satellites.. Ann Trop Med Parasitol.

[PDF_00661] Thomson M. C., Connor S. J., Milligan P., Flasse S. P. (1997). Mapping malaria risk in Africa: What can satellite data contribute?. Parasitol Today.

